# Male silver eels mature by swimming

**DOI:** 10.1186/1472-6793-8-14

**Published:** 2008-07-10

**Authors:** Arjan P Palstra, Denhi Schnabel, Maaike C Nieveen, Herman P Spaink, Guido EEJM van den Thillart

**Affiliations:** 1 Integrative Zoology, Institute of Biology, Leiden University (IBL), van der Klaauw Laboratory, Kaiserstraat 63, 2311 GP Leiden, The Netherlands; 2 Molecular Cell Biology, Institute of Biology, Leiden University (IBL), Clusius Laboratory, 2333 AL Leiden, The Netherlands

## Abstract

**Background:**

If European silver eels are prevented from reproductive migration, they remain in a prepubertal stage by dopaminergic inhibition of pituitary activity. Because this inhibition is likely a requirement for an extended female growth stage, we tested if it is sex-specific by subjecting both sexes to stimulation by GnRHa (Gonadotropin-Releasing Hormone agonist) – injection or 3-months swimming in seawater.

**Results:**

In contrast to females, males showed a two- to three-fold higher LHβ (luteinising hormone β subunit) – expression, a three- to five-fold higher GSI (Gonadosomatic index) and induced spermatogenesis when compared with the untreated control group.

**Conclusion:**

Dopaminergic inhibition is thus not effective in males and swimming results in natural maturation, probably via GnRH-release.

## Background

When European silver eels (*Anguilla anguilla*) venture in the ocean for their 5,500-km semelparous spawning run to the Sargasso Sea [1], they are still in a prepubertal stage. Sexual maturation has thus to occur during or after this long distance journey. Maturation in eels, as in other vertebrates, is regulated by the gonadotropic follicle-stimulating hormone (FSH) and luteinising hormone (LH) that are produced by the pituitary. If prevented from undertaking their oceanic migration, gonad development remains blocked by dopaminergic inhibition of pituitary activity as well as the absence of stimulation by Gonadotropin-Releasing Hormone (GnRH) [2]. Information about natural maturation is lacking, because migrating and spawning eels have never been caught near the spawning grounds. There is however an urgent need for an understanding of eel reproduction, because populations are collapsing on a global scale [3].

Investigations on eel reproduction have been mainly focussed on females. They stay 7 – 30 years in the freshwater before migration, in contrast to 4–9 years for males. As a consequence, females reach a ten-fold larger size than males at the onset of migration. The long female growth stage is likely required for a successful production of more than one million eggs, which at spawning time accounts for 40–60% of the body weight [4]. As the energy requirements for males are far less than those for females, it is possible that the observed dopaminergic inhibition is sex-specific. We have tested this hypothesis by subjecting male and female eels to a GnRH-agonist (GnRHa), specifically the commercial product Gonazon For Fish (Intervet), as well as to stimulation by long-term swimming in seawater (SW) that is supposed to stimulate GnRH excretion by the hypothalamus. Recently we found that swimming in freshwater (FW) triggers the enlargement of the eyes and development of oocytes in female eels [5,6]; all signs of early maturation, suggesting that swimming is a natural trigger for sexual maturation. However, further maturation (e.g. vitellogenesis) was not stimulated by FW-swimming, and may only be stimulated by SW-swimming during natural migration.

## Results

**Figure 1:**
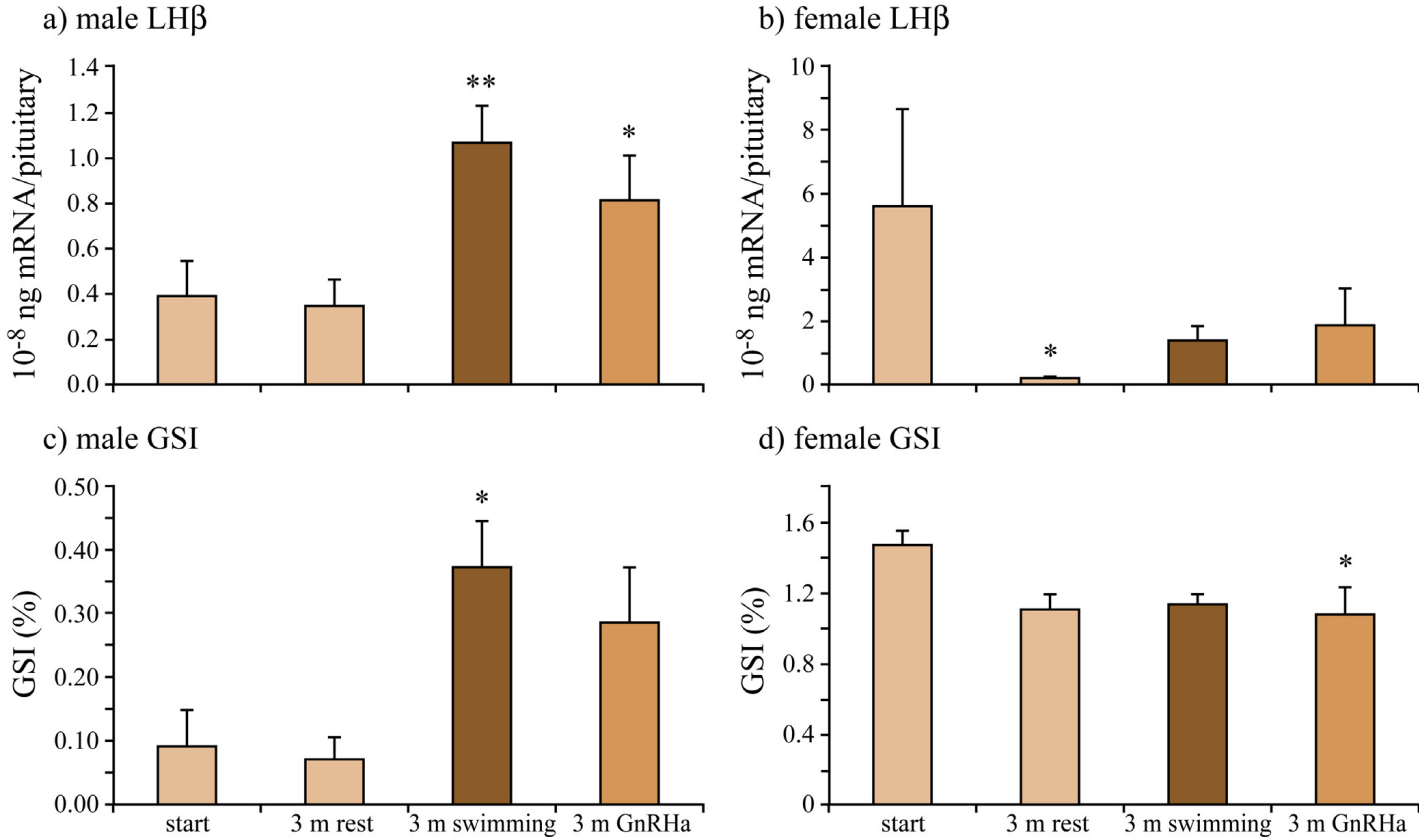
**Expression (Q-RT-PCR) of luteinising hormone subunit (LHβ) in the pituitary and the gonadosomatic index (GSI) in male and female eels**. Eels were sampled at the start, after three months rest, after three months of SW-swimming and three months after a single GnRHa injection (Gonazon For Fish, Intervet). In females, regression occurs during the experimental period, an effect which is more pronounced in the resting group than those stimulated by SW-swimming and GnRHa. In males however, SW-swimming and GnRHa activate maturation (student t-tests with * = P < 0.05; ** = P < 0.01; 3 m rest vs. start, 3 m swimming or 3 m GnRHa vs. 3 m rest).

**Figure 2:**
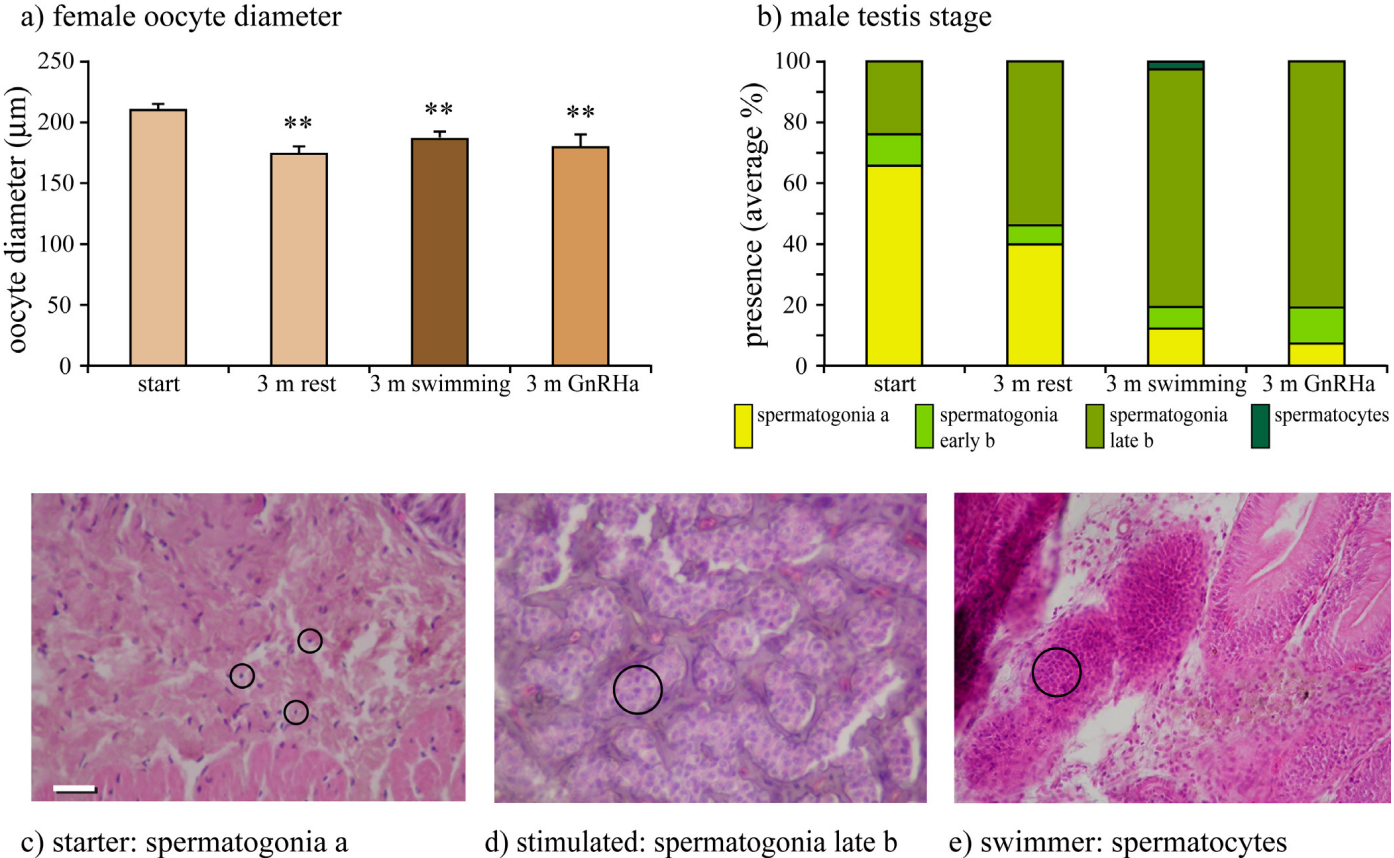
**Gonad development parameters in males and females**. a) oocyte diameters at the start, after 3 months of rest, after 3 months of SW-swimming and 3 months after GnRHa injection; b) male testis stage with frequency distribution of spermatogonia type a, spermatogonia early type b, spermatogonia late type b and spermatocytes; c) testis in starter containing mainly spermatogonia type a (encircled), d) GnRH-stimulated testis containing spermatogonia late type b (encircled), e) testis of swimmer containing spermatocytes (encircled). The scale bar represents 100 μm. Asterisks indicate significant differences (student t-test with ** = P < 0.01; 3 m rest, 3 m swimming or 3 m GnRHa vs. start).

Males that were either stimulated by three months SW-swimming or by GnRHa-injection showed a two- to three-fold higher LHβ expression level than the male starters and resters (Fig. 1a). Both treatments also caused a three- to five-fold increase in GSI (Fig. 1b) and an induced spermatogenesis (>80% presence of spermatogonia late type b; Fig. 2). One male swimmer even showed the formation of spermatocytes (Fig. 2). In contrast, females were not stimulated by SW-swimming nor by GnRHa, and even showed regression of maturation over time as demonstrated by lower LHβ expression (Fig. 1c), GSI (Fig. 1d) and oocyte diameters in all groups after 3 months (Fig. 2). The expression of FSHβ did not significantly change under the different treatments in both males and females.

## Discussion and conclusion

The results show an obvious difference between male and female eels in their response to both SW-swimming and GnRHa-injection. In female eels, GnRHa is not effective which confirms the conclusions by Dufour et al. [7] about prepubertal inhibition.

In contrast to the response of the females, we observed sexual maturation in males upon GnRHa-injection, indicating that dopaminergic inhibition is not effective in males. In addition we observed stimulation of sexual maturation in male eels after three months SW-swimming, suggesting that swimming acts via a similar mechanism. Swimming may up-regulate GnRH-levels (in silver eels the mammalian-type GnRH – mGnRH) that subsequently leads to positive effects on LHβ-production in the pituitary [8]. This action may be exerted through swimming-induced alterations in cortisol that binds to glucocorticoid receptor – expressing neurons [9]. However, in our study stimulation of LHβ-expression in the pituitary only occurred in male eels. Since LHβ-expression was not enhanced in females that either swam or received a GnRHa-injection, their pituitaries were considered as not sensitized and still under dopaminergic control.

As long-term swimming is required for reaching the spawning site, we conclude that swimming results in natural maturation in males, probably via the release of GnRH. Males swam in this experiment for about one sixth of their normal migration distance, so full spermiation can be expected after longer swimming trials. As naturally induced spermiation may result in improved sperm quality, we expect that swimming trials will improve the success of eel breeding.

## Methods

Migratory male (n = 28; 40 ± 0.5 cm, 96 ± 3 g) and female silver eels (n = 24; 75 ± 1 cm, 719 ± 38 g) were anaesthetized, PIT-tagged (TROVAN) and randomly divided over 4 groups of n = 6. Starters were immediately dissected (n = 10 for males). Other eels either swam or rested in natural seawater (35 ppt, 20°C) during the experimental period of three months. Resting eels were IP injected with GnRHa (32 μg/kg Gonazon) or left untreated ('resters') in a 1,500-l tank. An oval shaped stream-gutter (6.0*4.0*0.8 m; 6,000-l) was constructed to enable a 3-months swimming trial. Female eels swam 1,420 km while the smaller males swam 912 km. Pituitaries were stored in RNAlater (Ambion), mRNA was purified from these samples to quantify gene expression.

Oligonucleotides were designed from the reported sequences of *A. anguilla *for the specific β-subunits of LH and FSH, and of the Japanese eel *A. japonica *for housekeeping gene β-actin. A specific RT-PCR was performed using RNA extracts from pituitaries from artificially matured *A. anguilla *[4]. RT-PCR was performed using the Superscript II one step RT-PCR system with platinum Taq (Invitrogen), run on the Biometra T1 Thermocycler (Westburg). The PCR products were cloned in pCRII-TOPO vector (Invitrogen), digested with restriction enzymes to identify the correct direction and sequenced (Base Clear lab services) to verify that the final products correspond to the genes of interest by aligning them to genes previously described using the VectorNTI program.

Quantitative reverse transcriptase (Q-RT) – PCR using the MasterMix for SYBR^® ^Green I (Eurogenetec) and the Chromo4™ Detector (Bio-Rad laboratories) was performed to quantify the expression of LHβ, FSHβ, and of the internal control β-actin in the pituitaries. DNA and deduced mRNA amounts were calculated from Ct values of standard curves generated from the plasmids containing each of the specific genes after which amounts were normalized to the expression level of the housekeeping gene β-actin.

Experiments complied with the current laws of the Netherlands and were approved by the animal experimental commission (DEC nr. 6059).

## Abbreviations

FSHβ: Follicle-Stimulating Hormone β subunit; FW: Freshwater; GnRH: Gonadotropin-Releasing Hormone; GnRHa: Gonadotropin-Releasing Hormone agonist; GSI: Gonadosomatic Index; LHβ: Luteinising Hormone β subunit; SW: Seawater.

## Authors' contributions

APP and GvdT conceived and designed the project and the experiments. APP performed the experiments, measurements, dissection and histology. DS and HPS cloned β-actin and the LHβ and FSHβ subunits and tested the right conditions for Quantitative RT-PCR. MCN performed Quantitative RT-PCR. APP and GvdT wrote the paper.

## References

[B1] Schmidt J (1923). Breeding places and migration of the eel. Nature.

[B2] Vidal B, Pasqualini C, Le Belle N, Claire M, Holland H, Sbaihi M, Vernier P, Zohar Y, Dufour S (2004). Dopamine inhibits luteinizing hormone synthesis and release in the juvenile European eel: A neuroendocrine lock for the onset of puberty. Biol Reprod.

[B3] Stone R (2003). Freshwater eels are slip-sliding away. Science.

[B4] Palstra AP, Cohen EGH, Niemantsverdriet PRW, van Ginneken VJT, Thillart GEEJM van den (2005). Artificial maturation and reproduction of European silver eel: Development of oocytes during final maturation. Aquaculture.

[B5] van Ginneken V, Dufour S, Sbaihi M, Balm P, Noorlander K, de Bakker M, Doornbos J, Palstra A, Antonissen E, Mayer I, Thillart G van den (2007). Does a 5,500-km swim trial stimulate early sexual maturation in the European eel (*Anguilla anguilla *L.)?. Comp Biochem Physiol A.

[B6] Palstra A, Curiel D, Fekkes M, de Bakker M, Székely C, van Ginneken V, Thillart G van den (2007). Swimming stimulates oocyte development in European eel. Aquaculture.

[B7] Dufour S, Lopez E, Le Menn F, Le Belle N, Baloche S, Fontaine YA (1988). Stimulation of gonadotropin release and of ovarian development, by the administration of a gonadoliberin agonist and of dopamine antagonists, in female silver eel pretreated with estradiol. Gen Comp Endocr.

[B8] Montero M, Le Belle N, King JA, Mîllar RP, Dufour S (1995). Differential regulation of the two forms of gonadotropin-releasing hormone (mGnRH and cGnRH-II) by sex steroids in the European female silver eel (*Anguilla anguilla *L.). Neuroendocrinology.

[B9] Teitsma CA, Anglade I, Lethimonier C, Le Dréan G, Saliguat D, Ducouret B, Kah O (1999). Glucocorticoid receptor immunoreactivity in neurons and pituitary cells implicated in reproductive functions in rainbow trout: A double immunohistochemical study. Biol Reprod.

